# The Influence of Textile Substrates on the Performance of Textronic RFID Transponders

**DOI:** 10.3390/ma15207060

**Published:** 2022-10-11

**Authors:** Piotr Jankowski-Mihułowicz, Mariusz Węglarski, Bartłomiej Wilczkiewicz, Mateusz Chamera, Grzegorz Laskowski

**Affiliations:** 1 Department of Electronic and Telecommunications Systems, Rzeszów University of Technology, ul. Wincentego Pola 2, 35-959 Rzeszów, Poland; 2 Talkin’ Things, Al. Wilanowska 317, 02-665 Warsaw, Poland; 3 Research & Development Center of Esotiq & Henderson S.A., ul. Budowlanych 31c, 80-298 Gdańsk, Poland

**Keywords:** RFID technology, RFID transponder, sewn tag, embroidered tag, textronics, dielectric properties of textiles, dielectric constant, dielectric loss

## Abstract

Recent advances in the development of innovative textronic products are often related to the implementation of radio-frequency identification (RFID) technology. Such devices contain components of wireless telecommunications systems, in which radiofrequency circuits should be designed taking into account not only the frequency band or destined application, but also the dielectric properties of the materials. As is known from the theory of RFID systems, the dielectric permittivity and loss angle of the substrates significantly affect the performance of RFID transponders. Therefore, the knowledge on the variability of these parameters is highly important in the context of developing new solutions in textronic devices with the RFID interface. According to the plan of studies, at the beginning, the comprehensive characterization and determination of the dielectric parameters of various types of textile substrates were carried out. On this basis, the influence of fabrics on the performance of textronic RFID (RFIDtex) tags was characterized with numerical calculations. As the RFIDtex transponders proposed by the authors in the patent PL231291 have an outstanding design in which the antenna and the chip are located on physically separated substrates and are galvanically isolated, the special means had to be implemented when creating a numerical model. On the other hand, the great advantage of the developed construction was confirmed. Since the impedance at the chip’s terminals is primarily determined by the coupling system, the selected fabrics have relatively low impact on the efficiency of the RFIDtex transponder. Such an effect is impossible to achieve with classical designs of passive or semi-passive transponders. The correctness of the simulations was verified on the exemplary demonstrators, in threshold and rotation measurements performed at the laboratory stand.

## 1. Introduction

### 1.1. Motivation

The progress in radio-frequency identification (RFID) technology is mainly stimulated by the results of the latest research and developments carried out in various fields of science and economy. Interdisciplinarity is particularly evident in the branch of intelligent textiles [1,2], where efforts are being made to develop innovative textronic products. These devices often contain components of wireless telecommunications systems, in which radiofrequency (RF) circuits should be designed taking into account not only the frequency band or destined application, but also properties of the materials, technical means of production, etc. [3]. The accuracy of designs prepared for the textronic devices with the RFID interface depends in particular on the knowledge of the dielectric properties of the substrates/textiles [4,5]. These parameters, although important, are not of key importance in the case of multichannel/broadband textile antennas dedicated to classic radio communication systems with an impedance of 50 Ω [6,7]. The problem is much more important in the case of RFID systems of the UHF band (operating frequency *f*_0_ = 860–960 MHz) [8,9]. The input impedance of RF front-ends in RFID chips changes during the operation of the transponders, depending on the strength of the electromagnetic field or computing tasks performed in a digital circuit, and is affected by many factors, such as tag orientation, distance from antenna of read/write device (RWD), proximity of other objects or obstacles, interferences by other electromagnetic fields, etc. Moreover, the impedance is not equal to the real value but is expressed by complex number. The properties of materials used in tag structures also significantly affect the impedance. All these factors have great impact on the interrogation zone (IZ), which is the basic application parameter of RFID systems. As a consequence, the impedance matching between the antenna and the chip’s RF front-end is the crucial problem when designing new RFID transponders [10].

In the presented research, the characteristics and determination of the dielectric parameters (complex electric permittivity) of selected substrate materials were made in the context of their usefulness in the process of designing the proposed textronic RFIDtex transponder. The idea of the RFIDtex tag construction is presented in detail in reference to the contemporary state of the art in Section 1.2. Additionally in this section, the difference between designing a standard 50 Ω antenna on textile substrate and antennas for RFIDtex transponder is highlighted. In Section 1.3, some information on the variety of textile materials is given. Next (Section 1.4), dielectric parameters important in the context of the presented research are indicated. In Section 2.1, the preliminary measurements of the dielectric properties are described for different types of textile materials. These results are implemented in the real problem of designing an RFIDtex transponder, and then the correctness of the elaborated numerical calculations is confirmed by the experimental investigations (Section 2.2). In this part, the impact of the determined parameters’ variability on the performance of RFIDtex transponders is characterized as well. The final discussion is in Section 3.

### 1.2. State of the Art

**Figure 1:**
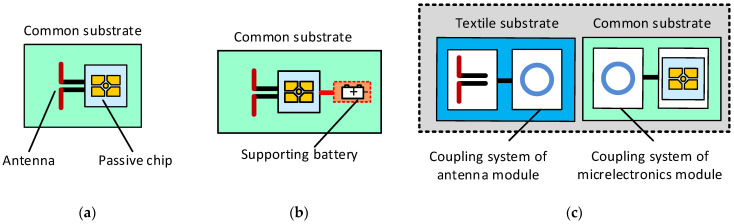
Models of RFID transponders: (**a**) passive transponder; (**b**) semi-passive transponder; (**c**) textronic transponder—RFIDtex tag.

The most common passive RFID transponder consists of a semiconductor chip and an antenna (Figure 1a). Access to the internal memory of the chip is possible only by a read/write device using radio waves [11]. Another group of growing market importance is semi-passive RFID transponders. An additional built-in power source (e.g., a removable or nonremovable lithium battery) distinguishes this group of devices (Figure 1b). In terms of construction, the concept of textronic RFID (RFIDtex) tags, proposed by the authors (Figure 1c) [12], stands out. In their structure, the antenna and chip are located on physically separated substrates and are galvanically isolated. The antenna module may be embroidered or sewn with conductive threads, as well as other techniques, e.g., by ironing/bonding metal wires into a textile. The microelectronic module with the chip is made as an electronic circuit on a printed circuit board (PCB) or flexible board, then is protected against environmental hazards, and is attached to the antenna coupling circuit by means available in the textile industry. Recent advancements in methods of manufacturing tags for marking wearable products are summarized in the review paper [13].

In recent years, the subject of antennas integrated with textile materials has become an area of intense interest of research centers around the world. In most cases, 50 Ω antennas dedicated to classic radiocommunication systems are considered in scientific publications or design studies. Some of them concern constructions that are dedicated to the unlicensed ISM (Industrial Scientific Medical) bands. Typically, these antennas are made by combining (a) classic conductive materials with a textile base (e.g., copper foil or screen-printed layer on cotton [14] and other substrates [15]) or (b) innovative conductive textile materials (e.g., conductive polymers, electrotextiles, graphene sheet, fabrics with metal threads [16,17]) with classic textiles (e.g., graphene sheet with denim or felt [13,18]). In addition, the integration of a typical flexible antenna (e.g., made on polyethylene terephthalate PET foils) with fabrics is frequently used [18,19]. The latest solutions are related to the increasing availability of numerically controlled machines and new materials. According to advances reported in the literature, the inductive structures are sewn or embroidered with conductive threads and then the integrated circuits (ICs) are bonded to them [20,21,22].

In the field of RFID technology, the first laboratory designs of sewn/embroidered passive transponder antennas can be noticed. The research is described in a few publications by Finnish and American researchers [9,23,24]. The classic/galvanic connection of the semiconductor chip with the antenna is used in the presented construction (the same as in [25,26]). Such a connection is vulnerable to the damaging effects of environmental hazards that occur throughout the life cycle of products. Moreover, the concept of such a textile RFID tag is, by definition, difficult to implement in processing lines of the textile industry [27] or, in the case of so-called washing tags (e.g., [28]), the separate process of product confectioning has to be provided.

Thus, the advances in the 50 Ω textile antennas, noticed in recent years, do not translate into research and development (R&D) works in the field of RFID technology. The problem is that both the operation principles of the RFID devices and the means of determining their parameters are different than those in classic radiocommunication systems. A huge challenge when designing standard RFID tag solutions is primarily the complex input impedance of the chip that changes during tag interrogating, its strong dependence on dielectric properties of the environment, and the need to match it to the antenna impedance [10]. The problem arises if we take into consideration the variety of materials (characterized in Section 1.3) in the case of textronic devices. This causes an increased risk of applications and disrupts dissemination of cheap and useful solutions for the IoT sector. The combination of the proposed concept of the RFIDtex tags with the possibility of defining and verifying their parameters significantly determines the process of synthesizing the interrogation zone in radio object identification systems.

### 1.3. Characteristics of Textile Substrates

The basic characteristics of textile substrates and a qualitative assessment of their properties were prepared on the basis of information commonly available in the literature [29,30].

The textile products (woven, knitted, nonwoven fabric) are obtained from yarns. The woven fabric is created as a result of interlacing mutually perpendicular systems of warp and weft threads, the knitted fabric is made of rows or columns of interconnected meshes, while nonwoven fabric (including felt) is a product made of a stream of felted, glued, needle-punched, or stitched fibers. In the textile industry, mainly five types of weaves are used, three of which concern woven fabrics (linen, satin, twill), and two knitted fabrics (weft and warp). They are commonly used in popular clothing materials that are considered as substrates for the structures of the RFIDtex tag.

Yarn is a semifinished product that is used in the production of fabrics. With respect to the types and properties of clothing materials, yarn fibers can be classified into the group of natural (plant–animal) or chemical origin. The first of them includes, among others, cotton, silk, and wool fibers, and the second—chemical fibers from natural, artificial, or synthetic polymers. Mixtures of items from both mentioned groups are also possible, which results in obtaining new properties of introduced fabrics.

Cotton is airy and comfortable to wear and touch. It has high hygroscopicity, retains heat, and dries slowly when wet. It is durable, not very elastic, and stretchy, it wrinkles strongly and is not prone to electrification. Silk is pleasant to the touch, absorbs moisture, and maintains a feeling of coolness, despite the fact that it retains heat well. This is a stretchy and elastic material, which means it does not crease too much. The linen fabrics remain cool to the touch, quickly absorb moisture, and release it into the environment just as quickly. With regard to cotton, linen is a harder, stiffer material and is therefore less pleasant to the touch. In the mechanical context, it has a very high strength, it is nonstretchable, not very elastic, and it bends strongly. The woolen materials are delicate, soft, and coarse. Wool is hygroscopic and although it easily absorbs moisture, it does not feel damp, which means that it neutralizes sweat and dries slowly. It is moderately durable and, at the same time, very stretchy and resilient.

In the second group of fabrics made of chemical fibers, the viscose materials have a structure comparable to that of cotton, but are more absorbent and pleasant to touch, which means that they have better functional properties. It should be emphasized that viscose is less durable, twice as stretchable, has little elasticity, and is subject to strong creases. Viscose fabrics can have different properties because they can be both thin and soft, as well as stiff, and they can also be shiny or matte. The group of artificial polymers includes polyacrylonitrile and modacrylic fibers. The first is similar to wool and silk. They have low density and high resistance to chemical compounds and light, and under the influence of high temperature they degrade (shrink, melt, and burn). They are usually used for the production of outerwear, imitation fur, and duvets, as well as upholstery and carpets. Modacrylic fibers inhibit fire spread, so they are used in the production of fireproof clothing. Polyamide and polyester synthetic fibers can take the form of yarns of various thickness and gloss. This material does not absorb moisture, is resistant to fungi and bacteria, has high mechanical strength, is stretchable and elastic, and fabrics made of these fibers do not crease. In terms of differences, polyester fibers are less stretchy than polyamide fibers.

It is obvious then that such a large variety of fibers and materials made of them also leads to the diversity of their dielectric parameters, which determine the properties of radio circuits.

### 1.4. Basic Parameters of Substrate

Determination of the relative electric permittivity (*ε_r_*) and the tangent of the loss angle (tg*δ*) in a given frequency band are necessary prior to performing the correct design of the required textronic structure in advanced numerical tools. These parameters should be obtained for the substrates of both: microelectronics and antenna module.

**Figure 2:**
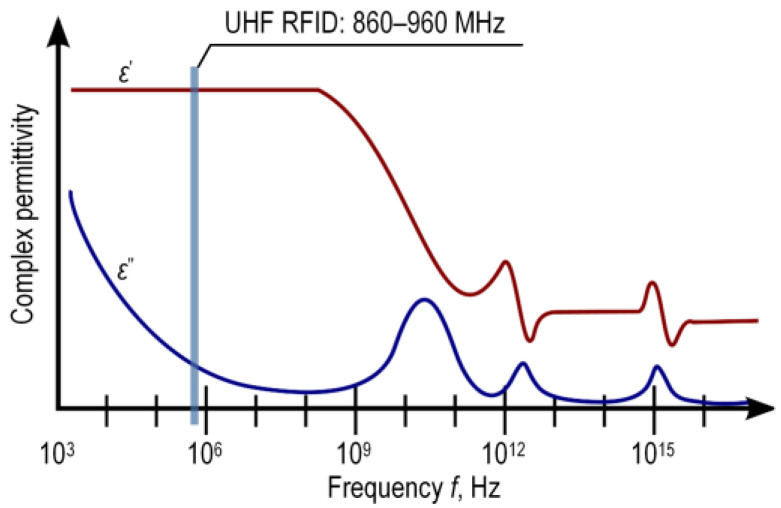
Dependence of complex permittivity on frequency, taking into account the UHF band of radio object identification systems.

The basic parameter of the substrates dedicated to the synthesis of textronic structures is the electric permittivity *ε*. In the classification by International Electrotechnical Commission IEC 80000-6: 2008 [31] (general information and definitions concerning quantities, systems of quantities, units, quantity and unit symbols, and coherent unit systems) and in scientific and technical literature, it is called dielectric permittivity. This material parameter strongly depends on the frequency of the electric field that affects the substrate (Figure 2). Thus, it is often treated as a complex function (1).

The complex electric permittivity of materials can be expressed by the following equation:

(1) $$\varepsilon =\varepsilon^{\prime }+j\varepsilon^{\prime \prime }=\varepsilon_{0}\varepsilon_{r}\left(1-j\;\mathrm{tg}\delta \right),$$

where *ε*′ means the real part, *ε*″ imagines part of permittivity, *ε*_0_ is vacuum permittivity (8.85 × 10^−12^ F/m), *ε_r_*—relative permittivity, and tg*δ*—loss angle tangent.

In the subject matter of the RFIDtex synthesis problem, the electric field is generated in the antenna array of the textronic transponder. Its influence causes the electrical polarization of the dielectric substrate and leads to losses. As a result, it affects the impedance matching of the antenna and the chip [32]. The wrong prediction of this phenomenon at the stage of transponder design reduces the geometrical size of the IZ and, thus, the operating range of the entire RFID system. Consequently, it may lead to the inability to implement the required processes of automatic object identification [10].

In the case of the construction with a heterogeneous dielectric substrate, the dielectric parameters of each component of the fabric layer must be determined. This problem of substrate inhomogeneity can be reduced to the dielectric system of the homogeneous layer. The equivalent of the electric permittivity can be expressed as a relationship [33]:

(2) $$\varepsilon_{eq}={\left(\sum_{n=1}^{N}\frac{t_{n}}{\varepsilon_{n}}\right)}^{-1}\cdot \left(\sum_{n=1}^{N}t_{n}\right),$$

where *ε_n_* means permittivity value and *t_n_*—thickness for *n-th* layer.

The use of relationship (2) can lead to simplification of numerical calculations, which may bring significant savings in time and computing resources during the synthesis of complex textronic structures with the RFID interface, especially when the RFIDtex tag is made as embroidered antenna on a nonwoven fabric, a lined fabric, etc.

## 2. Materials and Methods

### 2.1. Measurements of Substrate Properties

#### 2.1.1. Sample under Tests

According to the plan of experimental studies, the characteristics and determination of the relative dielectric permittivity (*ε_r_*) and the tangent of the loss angle (tg*δ*) were performed for two different groups of materials. Textile samples obtained from ready-made clothing products were used in the preliminary tests, whereas the main research was carried out on textiles intended for the clothing industry.

**Figure 3:**
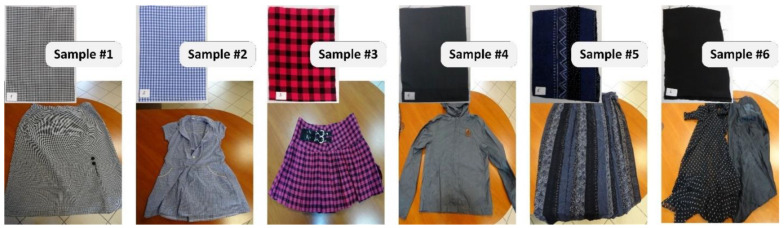
Test samples of the preliminary experiment.

The preliminary tests were carried out for six materials commonly used in the production of garments (Table 1). The samples were obtained from ready-made clothing (Figure 3).

The material composition was identified on the basis of information contained in the labels of the products.

The main research was carried out on an extended group of fabric substrates dedicated to the textile industry. Due to the large number of samples, the indexes were introduced according to the scheme: G*x*M*y*S*z*, where

- *x*—group of samples;
- *y*—type of fabric in the selected group of samples G*x*;
- *z*—number of the sample in the subgroup G*x*M*y*.

**Figure 4:**
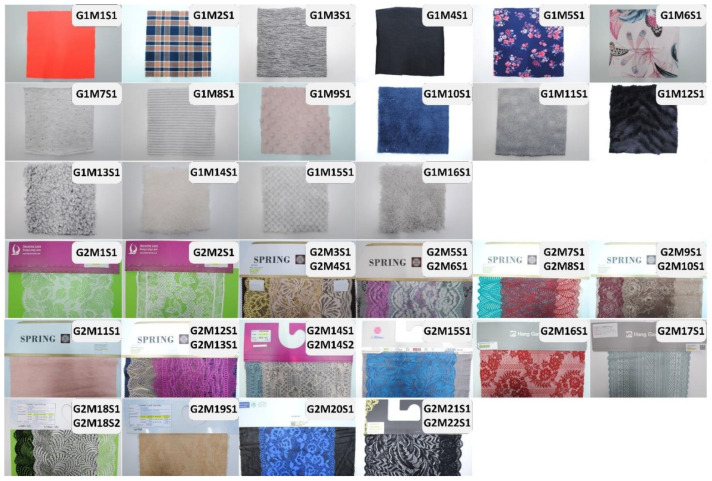
Test samples of the main experiment.

Five 15 × 15 cm samples were prepared from each material of the group G1 (Figure 4). Only one sample was made of materials from groups G2. The material composition was identified on the basis of the manufacturer’s data (Table 2).

#### 2.1.2. Measurements of Thickness

**Figure 5:**
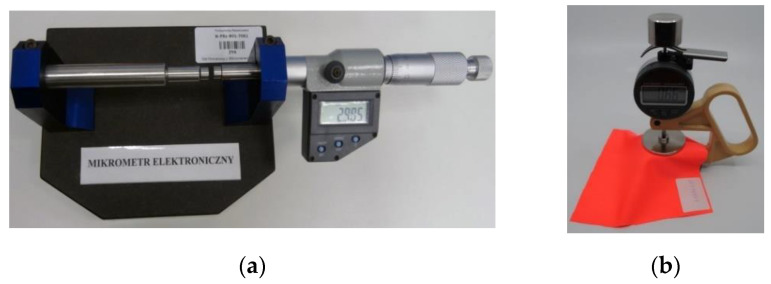
Measurement of substrate thickness: (**a**) preliminary experiment; (**b**) main experiment.

Correct determination of the textile sample thickness seems to be essential for the precise calculation of the dielectric properties. In the preliminary experiment, this parameter was determined with the use of simple and cheap equipment: a measuring table with an electronic micrometer Gimex 504.131 (Figure 5a). Measurements of the thickness were repeated five times (Table 3). The tightening force of the micrometer screw was intuitively adjusted so as not to deform the sample thickness. On the basis of obtained results, the average values were determined for six preliminary samples, #1–#6.

Textile substrates usually have a delicate structure, and even a slight pressure changes their thickness. This is due not only to the properties of the yarn and fibers, but also to the weave type. Therefore, instruments that conform to the requirements of International Organization for Standardization ISO 5084: 1996 [34] should be used to determine the thickness of flat fabrics.

In the main experiment, the thickness of the prepared samples G1M1S*z*–G2M22S1 was determined using the Checkline J-40-T portable digital gauge (Figure 5b), which complies with the ISO 5084: 1996 standard with regard to the surface of the foot and its clamping force (measuring range 0–10 mm, resolution 0.01 mm, flat foot diameter 50.42 mm, area 20 cm^2^, pressure 1 kPa). This device cannot be used for textile floor coverings, nonwoven fabrics, geotextiles, and coated fabrics.

Measurements were carried out for each of the prepared samples while maintaining the same pressure force of the thickness gauge on the tested substrate (Table 4). The average value of the thickness was calculated from five successively tested samples (S1–S5), for each material of the first group (G1M1S*z*–G1M16S*z*). In the case of the G2 group, there was only a single sample for a given material (G2M*z*).

#### 2.1.3. Measurements of Dielectric Permittivity

**Figure 6:**
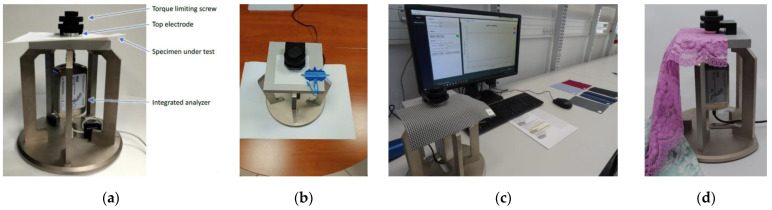
Measurement of dielectric permittivity: (**a**) epsilometer; (**b**) calibration of the measurement apparatus; (**c**) testbed; (**d**) sample testing.

In the final stage of the preliminary experiment, the relative dielectric permittivity (*ε_r_*) and the tangent of loss angle (tg*δ*) of the prepared samples with numbers #1–#6 were determined. These measurements were made with the use of the epsilometer system [35], consisting of the Compass Technology measuring device (Figure 6a), Vector Network Analyzer (VNA) Copper Mountain R60, and a dedicated software package for processing gathered data (Figure 6c). The parameters were obtained in the range of 1 MHz to 6 GHz.

**Figure 7:**
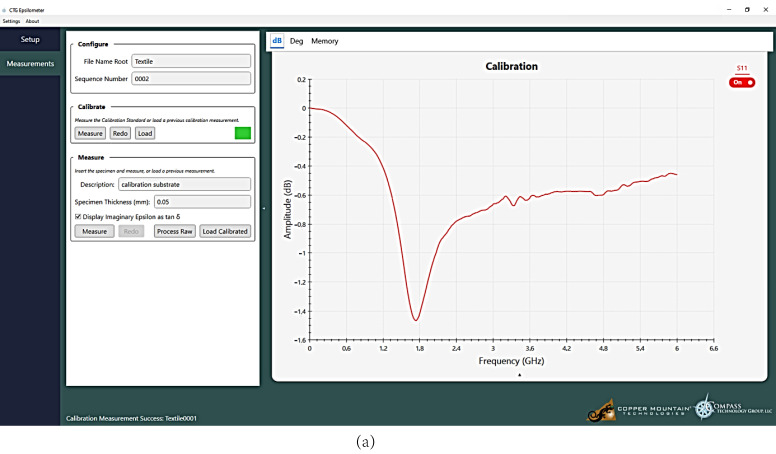

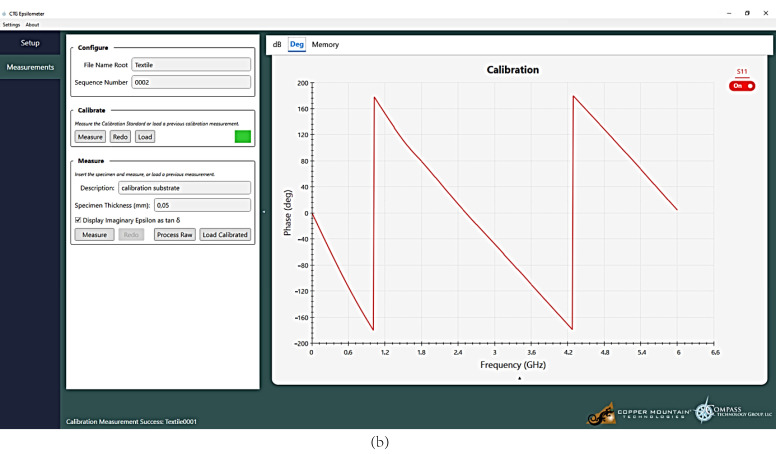
Results of apparatus calibration: (**a**) amplitude characteristic; (**b**) phase characteristic.

The measurement process started with the calibration of the test stand (Figure 6b). It was carried out with the use of a dedicated Teflon calibrator [36]. The amplitude and phase characteristics were obtained as a result of this process (Figure 7). They were used as the reference point for the measurements performed.

**Figure 8:**
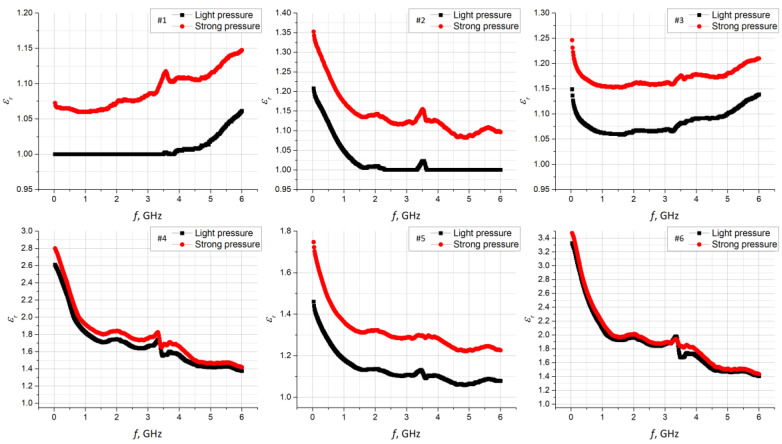
Comparison of *ε_r_* for light and strong pressure on substrate samples.

The average values of the substrate thickness obtained in the previous stage of the experiment were used to finally determine the complex electrical permittivity (Figure 8). These parameters were calculated on the basis of the model described by (1) dependence. The model expresses the unique design of the Compass Technology measuring device.

**Figure 9:**
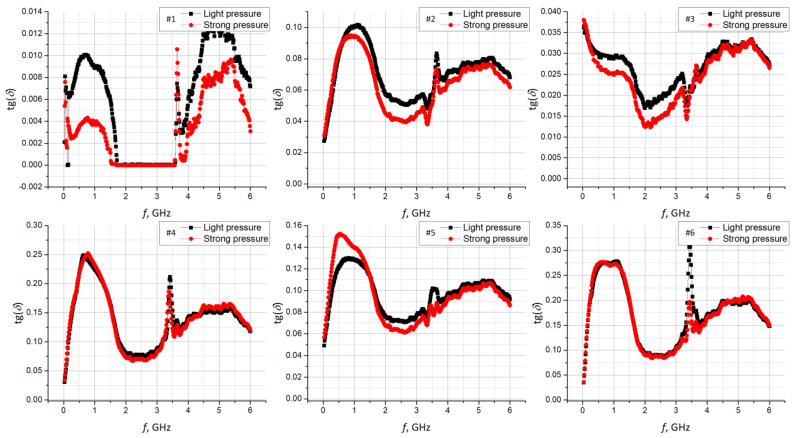
Comparison of tg*δ* for light and strong pressure on substrate samples.

Unfortunately, the epsilometer is not equipped with an indicator that could show the pressure force of the measuring head. Therefore, measurements were repeated for two tension levels of the top screw, that were designated as light and strong. The pressure was intuitively adjusted to avoid deforming the structure of the tested materials. As a result, the characteristics of the relative electrical permittivity and the tangent of the loss angle were obtained. They were determined for each sample #1–#6 in the frequency band of 1 MHz to 6 GHz, for two cases of clamping force: light and strong (Figure 8 and Figure 9). A statistical analysis was performed for the results presented. The Pearson coefficient was calculated between the light and strong pressure of each sample (Table 5).

The average value of the difference between the light and strong pressure of the samples in the measuring device was also determined for both dielectric parameters (Table 6).

The results obtained from the statistical analysis show a strong correlation. Thus, the uncertainty resulting from the applied method of determining the substrate parameters can be accepted for the synthesis of the textronic structures with the RFID interface.

**Figure 10:**
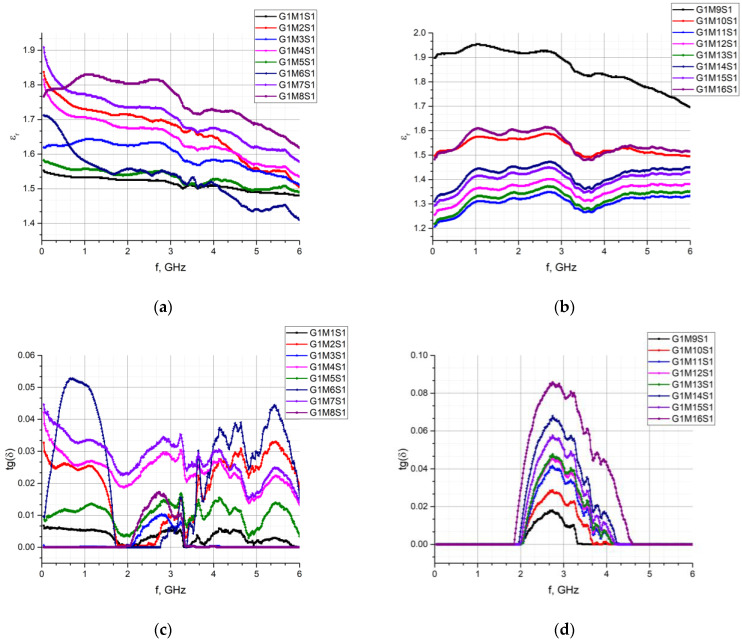
Comparison of dielectric parameters obtained in G1 group of fabrics: (**a**) *ε_r_* for G1M1S1–G1M8S1; (**b**) *ε_r_* for G1M9S1–G1M16S1; (**c**) tg*δ* for G1M1S1–G1M8S1; (**d**) tg*δ* for G1M9S1–G1M16S1.

**Figure 11:**
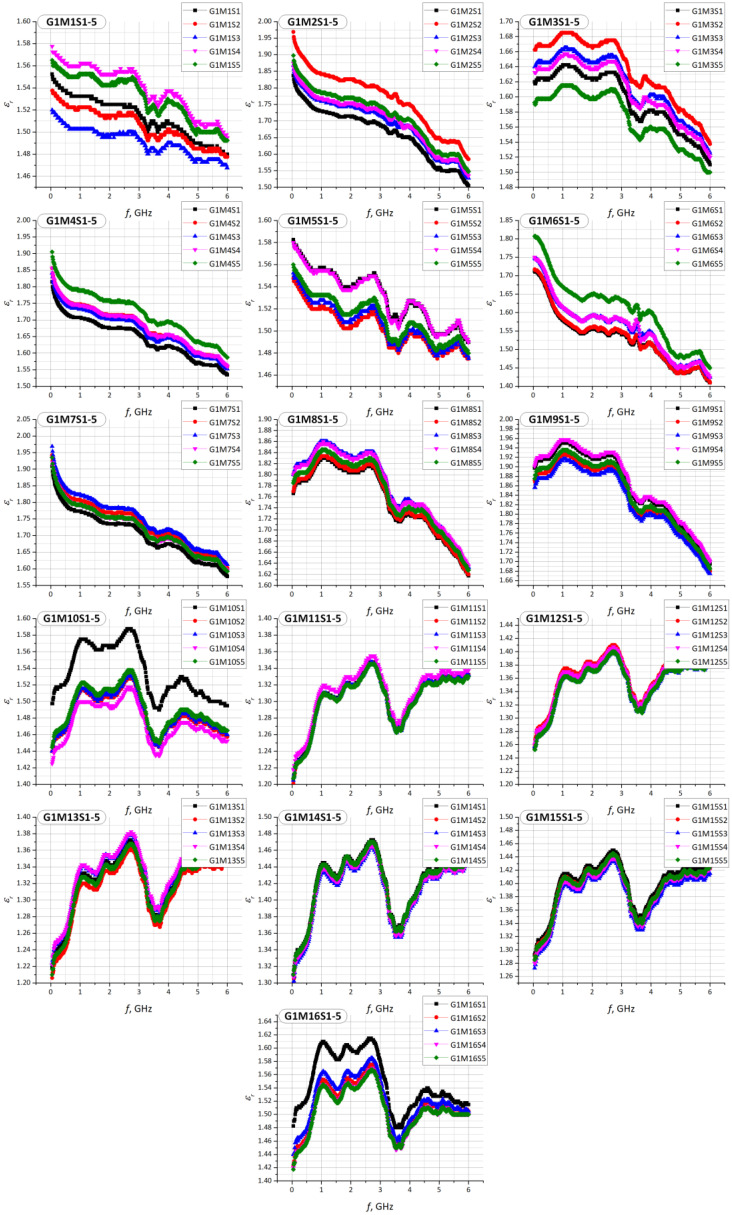
Results of *ε_r_* measurements for every sample of S1–S5 in G1 (G1M1S*z*–G1M16S*z*) group.

**Figure 12:**
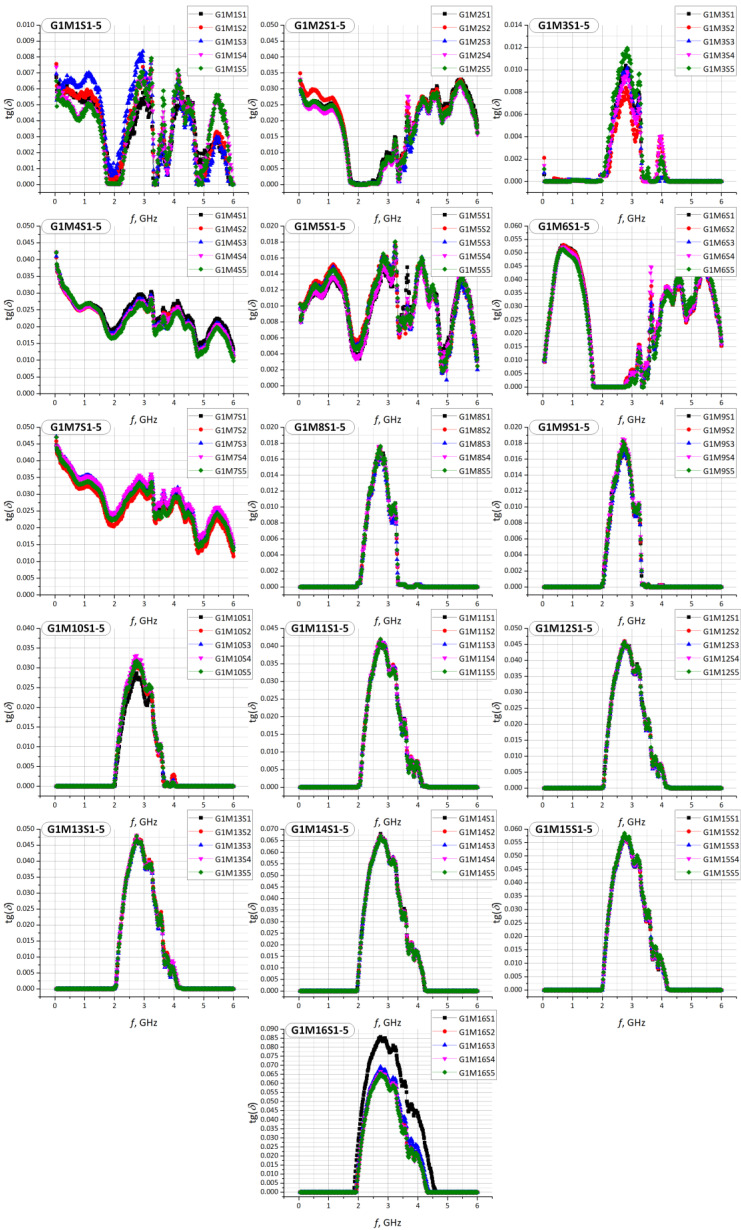
Results of tg*δ* measurements for every sample of S1–S5 in G1 (G1M1S*z*–G1M16S*z*) group.

**Figure 13:**
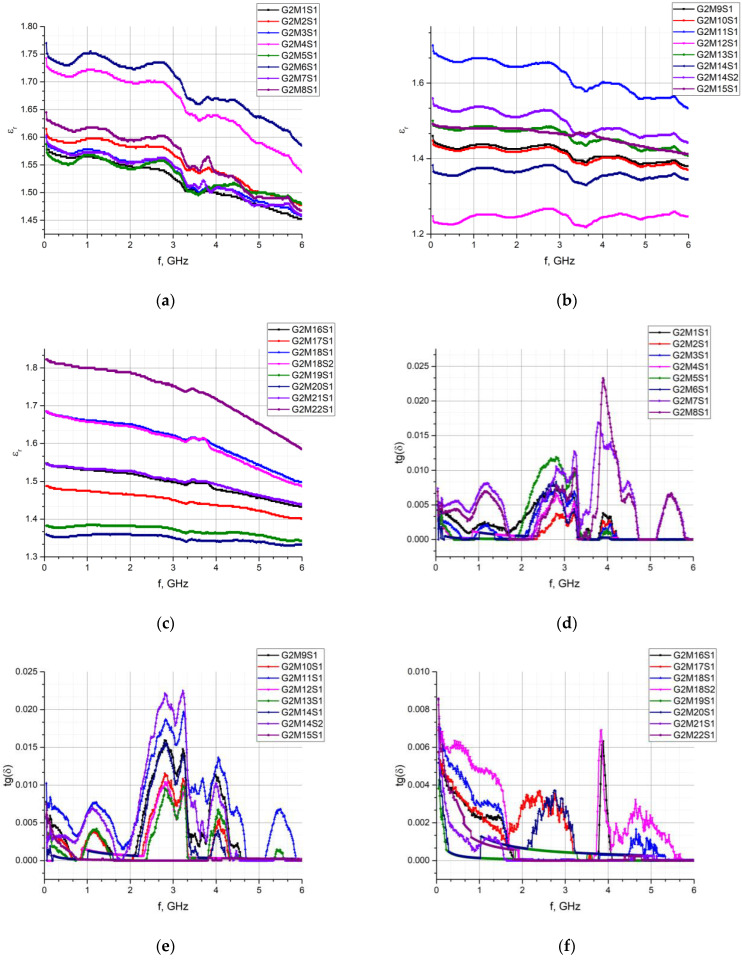
Comparison of dielectric parameters obtained in G2 group of fabrics: (**a**) *ε_r_* for G2M1S1–G2M8S1; (**b**) *ε_r_* for G2M9S1–G2M15S1; (**c**) *ε_r_* for G2M16S1–G2M22S1; (**d**) tg*δ* for G2M1S1–G2M8S1; (**e**) tg*δ* for G2M9S1–G2M15S1; (**f**) tg*δ* for G2M16S1–G2M22S1.

Similar experiments were performed for each sample G1M1S1–G2M22S1 and then the characteristics of the relative permittivity and the loss angle tangent were determined in the band of 1 MHz to 6 GHz. The results of the measurements are summarized in Figure 10, Figure 11, Figure 12 and Figure 13.

A statistical analysis was also performed for the results of this stage. The Pearson coefficient was calculated for each series of five samples of materials in the G1 group (G1M1S*z*–G1M16S*z*) and for each type of lace from the G2 group (G2M1S1–G2M22S1) It was determined in the whole range of the frequency band from 1 MHz to 6 GHz (Table 7).

The observed differences in the determined vales of the electric permittivity and the tangent of the loss angle (Figure 10, Figure 11 and Figure 12) result from the aforementioned inability to control the clamping force of the Compass Technology epsilometer head. However, statistical analysis shows a strong correlation between samples of the same material in the G1 group. This fact allows to accept the uncertainty resulting from the applied method of determining the substrate parameters. As it is shown in the next section, the results obtained in this way are sufficient for the efficient synthesis of electronic transponders according to the RFIDtex conception.

A comparison of different lace fabrics (G2M1S1–G2M22S1), especially in the loss angle tangent, gives a weak correlation. This is due to the significant heterogeneity in the thickness and structure of these types of materials. However, this is not critical as the multilayer substrate structure is typically used in the RFIDtex transponders.

The material properties of textiles under consideration were determined in a very wide frequency band up to 6 GHz. This was performed just for future investigations as well as in the context of conducting further, more extensive scientific studies and allowing comparisons of research results. In the context of the RFIDtex systems, the operation band (Figure 2) is confined to the range of 860–960 MHz. In this frequency range, the problem of the variability of the substrate parameters can be considered as quasi-stationary for a given textile. The problem may arise when the broader band of frequency is to be analyzed as well as when new composite material is considered [13,18,37,38].

### 2.2. Influence of Substrates on the Performance of RFIDtex Transponders

#### 2.2.1. Design of RFIDtex Transponder

The next step of the presented research and development work is to study the influence of the substrate materials on the parameters of the RFIDtex tag. The calculations performed were based on the dielectric parameters measured in the previous stage. A series of numerical calculations were carried out to show changes in the RFIDtex transponder impedance depending on the fabric used.

**Figure 14:**
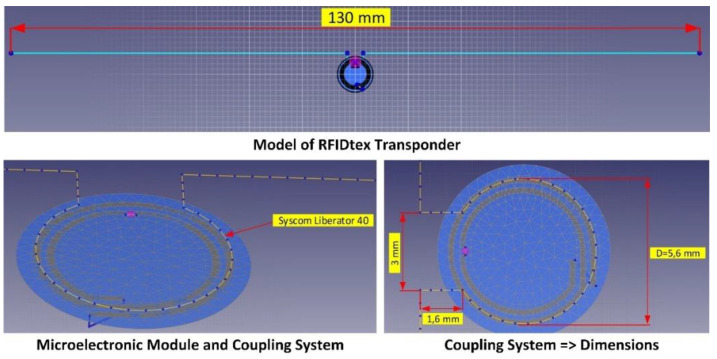
Numerical model of the antenna with microelectronic module (Syscom Liberator 40—conductive thread of antenna).

The model of the RFIDtex tag, built on the basis of the authors’ patent “*Textronic RFID transponder*” (Patent Office of the Republic of Poland No. PL231291), was used for the tests. A classical dipole antenna with a length slightly shorter than half the wavelength of 866 MHz was used in the design. The dipole was equipped with an additional loop that enables coupling with the microelectronic module (Figure 14). Numerical calculations were performed using the EMCoS Studio software tool.

#### 2.2.2. Input Data to Numerical Model

The NXP Semiconductors Ucode 7m (SL3S1214) chip [39] was used to design the RFIDtex transponder. The IC in the SOT886 housing (SL3S1214FTB0, XSON6) was implemented in order to facilitate the manufacturing of the microelectronic module.

The most important parameters (from the point of view of the RFID antenna designer) of the RFID chip are presented in Table 8.

The physical parameters of the substrate used to fabricate the microelectronic module were taken into account in the numerical model. The electronic circuit was made on the Du-PONT Pyralux LF9150R [40]. It is a flexible laminate made of a polyamide dielectric—Kapton, covered with a layer of copper with a thickness of 35 µm. In order to obtain the exact dielectric parameters of the material, measurements were made using the epsilometer system mentioned in Section 2.1.3. The relative dielectric permittivity and the tangent of the loss angle were determined in the range of 1 MHz to 6 GHz. The measured dielectric parameters at 866 MHz are shown in Table 9.

Additionally, the zero-ohm resistor was introduced into the demonstrator of the RFIDtex tag as a bridge connecting the loops of the coupling system. In the case of the numerical model, the coils were short-circuited, thus simulating the resistor used.

The impedance of the microelectronic module with the coupling system was adjusted to match the input impedance of the NXP Ucode 7m chip. As a result, the diameter of the outer loop was designated as 5.7 mm. The real and imaginary parts of the microelectronic module impedance are shown in Table 10.

A PACKLitzWire 10 × 0.04 mm [41] litz wire was used as a conductive thread to embroider the antenna. The litz wire used is a braided copper conductor, consisting of ten copper strands in a double silk insulation. Its basic parameters are presented in Table 11. A wire with a total thickness equal to the sum of the diameters of the copper strands and the equivalent of double silk insulation was introduced in the numerical calculations.

The relative dielectric permittivity of silk ranges from 2.5 to 3.5. The middle value *ε_r_* = 3 was assumed in the numerical calculations.

#### 2.2.3. Analysis of Substrate Impact

The influence of textiles was analyzed on the basis of the numerical model elaborated for the RFIDtex transponder in which there is no galvanic connection between the antenna and the chip. The parameters of the substrates that were considered in calculations are summarized in Table 12.

**Figure 15:**
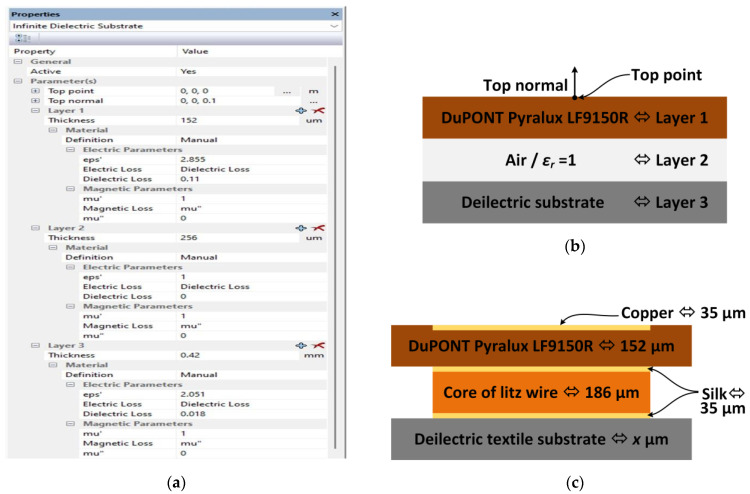
Model of RFIDtex transponder in EMCoS Studio: (**a**) definition of infinitive dielectric substrates; (**b**) model of dielectric substrates; (**c**) model completed with conductive components.

The multilayer structure of the substrate poses a problem in modeling. Its heterogeneity is reduced to the form of a layer-uniform system considered on the whole as the object affected by electromagnetic waves (free space, Kapton as a dielectric substrate for microelectronic module, textiles as dielectric carrier base for the antenna, and conductive objects: copper for tracks in microelectronic module and conductive thread for the antenna radiator). Due to the available options of the ECMCoS Studio software, it is not possible to create two finite dielectric substrates. Thus, they were defined as infinitive. A fragment of the program window along with the model visualization is shown in Figure 15. Each material used was assigned as a separate layer. A layer of air with the relative permittivity equal to 1 was placed between the dielectric textile substrate and DuPONT Pyralux in order to complete the model (Figure 15b). The dielectric model was supplemented with conductive components (Figure 15c). The litz thread is assumed to be on the surface of the fabric; thus, it is located in the air space. Due to the extensive research carried out so far, the influence of electrical parameters and the structure of conductive threads (Adafruit 603, 640 and 641, Sparkfun DEV-11791, Kitronik Electro-Fashion, Syscom Liberator 40 and Agsis) will be the aim of another publication.

**Figure 16:**
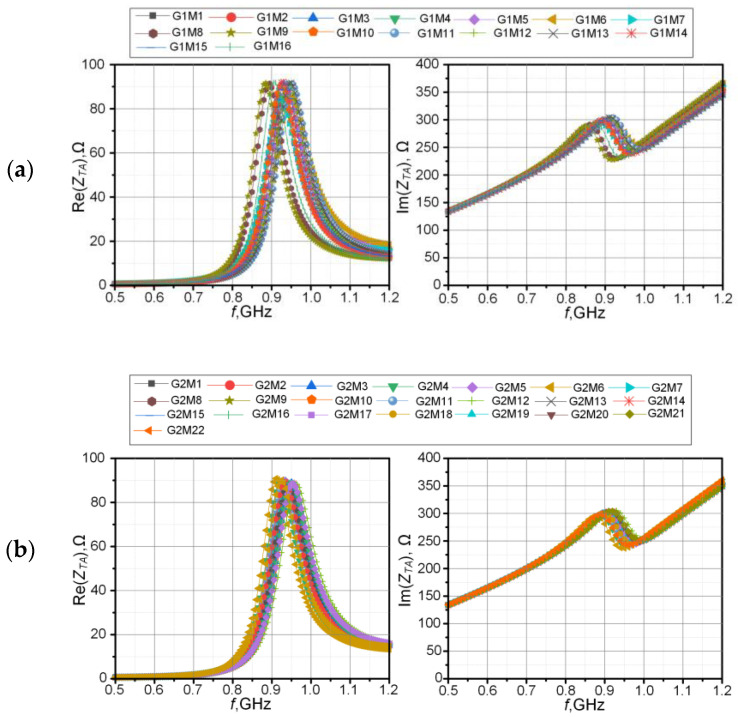
Complex impedance of RFIDtex transponder antenna depending on textile substrate: (**a**) Group G1; (**b**) Group G2.

**Figure 17:**
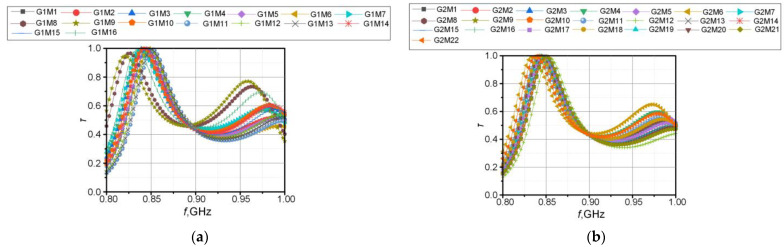
Power transfer coefficient complex impedance of RFIDtex transponder antenna depending on textile substrate: (**a**) Group G1; (**b**) Group G2.

In the beginning, the impedance parameters of the antenna were determined for the defined textile substrates. The real and imaginary parts of the complex impedance are shown in Figure 16. The charts were divided into two groups according to the type of fabric: G1 or G2. A comparison of the power transfer coefficient *τ* is presented in Figure 17.

The simulations performed show the influence of textile substrates used on the impedance parameters of the RFIDtex transponders. On the basis of the graphs, it can be observed that the selected fabrics have a relatively low impact on the impedance parameters of the antenna. The shapes of the graphs of the real and imaginary parts of the complex impedance do not change; only the self-resonance slightly shifts. The greatest difference can be observed for the G1M8 and G1M9 fabrics. The composition of these textiles is as follows: 100% polyester, 220 gsm and 100% polyester, 260 gsm. For these materials, the largest change in the parameter *τ* can also be observed. The maximum value of the power transfer coefficient shifts in the frequency axis by about 25 MHz to the left of the axis.

Particularly interesting results were achieved in the G2 group. According to Section 2.1.3, the measurements of the dielectric parameters resulted in a large dispersion of the obtained values due to the heterogeneity of laces. The problem seems to be serious as it is commonly assumed that the influence of substrate parameters, especially in terms of the relative dielectric loss variability, on the performance of a classical RFID transponder is a key determinant of an efficient identification system. However, we can see that for changes in the *ε*_r_ parameter (from the value 1.24 for G2M12 to 1.8 for G2M22), the power transfer coefficient only shifts by about 25 MHz. This is due to the structure of the RFIDtex transponder (Figure 1c). It is just insensitive to changes in the parameters of textile substrates. Such an achievement is not possible in classic RFID transponder constructions, where chips are galvanically connected to antenna terminals (Figure 1a).

#### 2.2.4. Verification of Numerical Calculations

In the verification stage, the basic parameters of the prepared RFIDtex demonstrators were determined. In this regard, the energy and data transmission in the system were characterized in the threshold measurements, as well as their changes in a given direction in the rotation measurements. On this basis, the operating range (read range) of the tag for data reading and energy transfer in an exemplary RFID system was determined.

**Figure 18:**
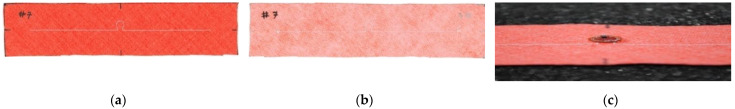
An example of the RFIDtex transponder—sample A7: (**a**) front of antenna; (**b**) back of antenna; (**c**) antenna and microelectronic module.

Seven demonstrators were made on the basis of the prepared design using a BROTHER INNOV-IS V3 embroidery machine (Figure 18). The measurements were made for samples with a microelectronic module attached (Figure 18c).

**Figure 19:**
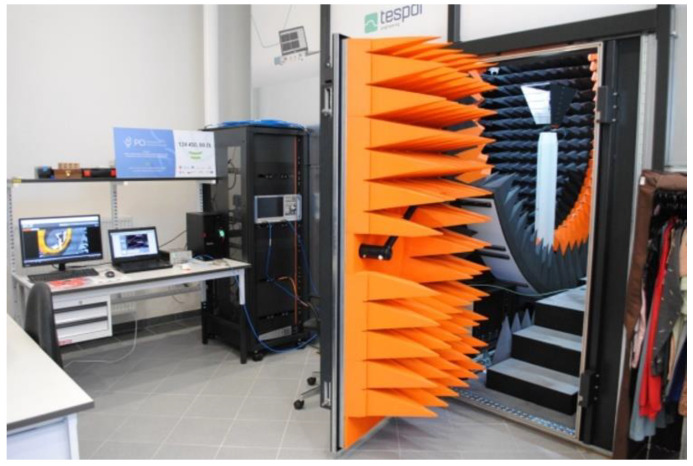
Laboratory experimental stand.

Measurements were made using the Voyantic Tagformance Pro system with Tagformance UHF v.13.2.3 software, as well as a set of RF equipment, which included 50 Ω directional coupler (frequency band: 600–1300 MHz, TX-ANT insertion loss: 1.2 dB, ANT-RX insertion loss: 6.5 dB, maximum input power: 250 W), broadband logarithmic-periodic antenna—AARONIA HyperLOG 7025 (frequency band: 700 MHz–2.5 GHz, VSWR < 2, typical 4 dBi gain), Florida RF Labs set of flexible test cables, and other RF components. The Microwave Vision Group anechoic chamber was used in the experiment (Figure 19).

The parameters of the ISO/IEC 18000-63 communication protocol, compliant with EPC Class 1 Gen2, configured during the measurements, are presented in Table 13.

The threshold measurements were carried out in the 800–1000 MHz frequency band, taking into account the RFID systems compliant with the requirements of the European Telecommunications Standards Institute (European version: ETSI EN 302 208, frequency band 865.6–867.6 MHz, 2 W ERP) and the Federal Communications Commission (US version: FCC Part 15.247, frequency band 902–928 MHz, 4 W EIRP (effective isotopically radiated power), 1 W output power of transmitter with antenna with a maximum gain of 6 dBi).

**Figure 20:**
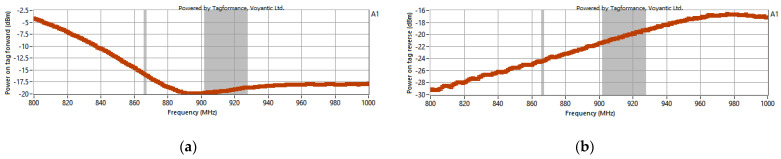
Exemplary sensitivity (for A1 sample) of energy (**a**) and data (**b**) transmission on RFIDtex tag.

The sensitivity of the transponder was measured for energy transfer (exemplary results of *P_Pwr_* for sample A1 are shown in Figure 20) and data transmission (*P_Btr_*—Figure 20b).

**Figure 21:**
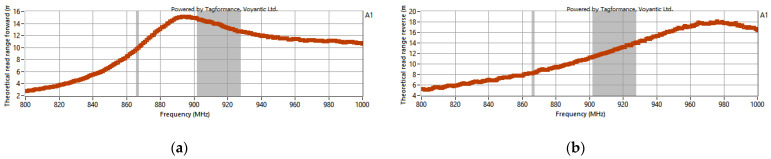
Exemplary range (for A1 sample) of energy (**a**) and data (**b**) transmission on RFID system.

As a result, the range was determined for energy transmission (forward link)—*r_PwrMax_* (measurement for A1 sample: Figure 21a) and data transmission (return link)—*r_BtrMax_* (measurement for sample A1: Figure 21b). The frequency bands of 865.6–867.6 MHz for the European standard and 902–928 MHz for the American standard are marked in gray in the charts.

**Figure 22:**
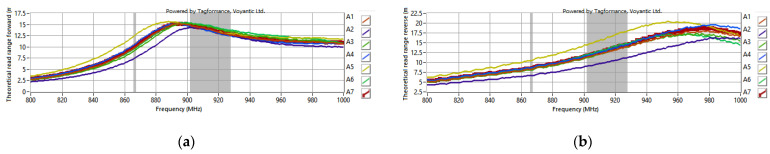
A1–A7 samples: range of energy (**a**) and data (**b**) transmission in RFID system.

Measurements were repeated for all seven prepared demonstrators of the RFIDtex transponder (Figure 22).

## 3. Results

The considerations on the dielectric properties of textile substrates were finally used in designing the UHF RFIDtex textronic transponder. On the basis of obtained results, it was possible to reach useful construction of the RFIDtex demonstrator which exposes the read range of several meters; assuming work in a system compliant with the requirements of ETSI EN 302 208 and EPCglobal: *P_EIRPmax_* = 3.28 W EIRP (35.16 dBm), *P_Rmin_* = 40 pW (−74 dBm). The performance of the elaborated device (in which the PACKLitzWire 10 × 0.04 thread was used to embroider antenna) is comparable to the design achieved in [42] but with the use of thread with 275 stainless steel filaments.

The performed numerical calculations allow the authors to conclude that no significant influence on the antenna resistance and reactance is observed for the tested samples of textile materials. Even if the dielectric parameters of the textile substrate exhibit large dispersion of the obtained values, especially in the G2 group, the power transfer coefficient for the RFIDtex transponder only shifts by about 25 MHz. Accordingly, it was found that it would be possible to use a single-antenna geometry when applied to different types of garments (pants, T-shirts, underwear, etc.). Such an achievement is not possible in classical RFID tag constructions, where chips are galvanically connected to antenna terminals. Thus, the authors reached the construction that is insensitive to changes in the parameters of textile substrates.

This achievement is important for several reasons. The universal design of the RFID tag dedicated for a variety of clothes (in terms of their type and materials used) can be easily accepted by the textile industry, as it is possible to implement it easily and efficiently (e.g., in the form of a washing tag). The developed design can be made immediately by sewing methods known in the garment industry. The RFIDtex transponder has predictable performance parameters (including, in particular, the range of the RFID system), regardless of the type of tagged product. This is because the impedance of its antenna is primarily determined by the coupling system of the microelectronics module and the antenna module. The radiator and its part of the coupling system in the antenna module just determine the proper waveform matching. Such an effect is impossible to achieve in the case of classical design of passive transponders (Figure 1a). Since the substrate properties strongly affect the antenna impedance, which significantly changes the power transfer coefficient and, consequently, the operating range of the RFID system, the tag’s construction has to be modified when the tagged product is changed. Moreover, the standard semi-passive transponder can only be used in logistic processes (e.g., in trade, Decathlon). It cannot be used throughout the life cycle of textile products [12], e.g., during washing or ironing. In this context, the obtained research results were the basis for the authors to start the process of creating a useful design of the RFIDtex tag for various products of the textile industry.

The material properties of textiles were determined in a very wide frequency band. This was performed in the context of conducting further, more extensive scientific studies. An interesting task is to design a transponder operating simultaneously in two standards (European 865.6–867.6 MHz and American 902–928 MHz) or on a substrate made of multilayer or composite fabric, e.g., covered with metal or ferromagnetic coatings. Moreover, due to the extensive research carried out so far, the influence of electrical parameters and the structure of conductive threads were omitted. This problem will be the aim of another publication.

Finally, it should be mentioned that the used ellipsometer does not have a clamping force indicator. Therefore, measurements were repeated for two levels of tensioning the top screw: light and strong. The pressure was intuitively adjusted to avoid deforming the structure of the tested materials. On the basis of the presented results, the obtained uncertainty can be accepted for the synthesis of the textronic structures with the RFID interface. In the case of a classical construction of an RFID tag, such an approach would not be possible. In this context, the possibility of extending the Compass Technology measuring device with the function of electronic pressure force control can be welcomed. Such a facility can be found, for example, in advanced control and measurement equipment for determining the thickness of textile materials [43] or complete systems for measuring dielectric parameters [44].

## Figures and Tables

**Table 1:** Set of material samples for preliminary experiment.

Sample No.	Material Composition	Kind of Garment
#1	100% polyester	Skirt
#2	60% cotton, 40% polyester	Dress
#3	100% cotton	Skirt
#4	100% nylon	Nylon jacket
#5	100% viscose	Skirt
#6	95% polyester, 5% viscose	Dress

**Table 2:** Set of material samples for main experiment.

No.	Index	Material Composition	No.	Index	Material Composition
1	G1M1S1	82% polyester 18% spandex	17	G2M1S1	85% nylon 15% spandex
2	G1M2S1	100% cotton 110 gsm	18	G2M2S1	92% nylon 8% spandex
3	G1M3S1	97% polyester 3% spandex	19	G2M3S1	91.5% nylon 8.5% spandex (yellow)
4	G1M4S1	95% cotton 5% spandex	20	G2M4S1	89.8% nylon 10.2% spandex (pink)
5	G1M5S1	80% polyamide 20% spandex	21	G2M5S1	90% nylon 10% spandex (pink)
6	G1M6S1	95% polyester 5% spandex	22	G2M6S1	85% nylon 10% spandex + 5% (green)
7	G1M7S1	99% cotton 1% viscose	23	G2M7S1	90% nylon 10% spandex (blue)
8	G1M8S1	100% polyester 220 gsm	24	G2M8S1	90% nylon 10% spandex (red)
9	G1M9S1	100% polyester 260 gsm	25	G2M9S1	81% nylon 19% spandex (claret)
10	G1M10S1	100% polyester (navy blue)	26	G2M10S1	88.5% nylon 11.5% spandex (beige)
11	G1M11S1	100% polyester (gray)	27	G2M11S1	75% nylon 25% nylon (pink)
12	G1M12S1	100% polyester (black)	28	G2M12S1	88.5% nylon 11.5% spandex (beige)
13	G1M13S1	100% polyester (gray)	29	G2M13S1	87.6% nylon 12.4% spandex (pink)
14	G1M14S1	100% polyester (white)	30	G2M14S1	85% nylon 15% spandex (blue)
15	G1M15S1	100% polyester 280 gsm	31	G2M14S2	85% nylon 15% spandex (creamy)
16	G1M16S1	92% polyester 8% metallized thread	32	G2M15S1	84% nylon 16% spandex (blue)
			33	G2M16S1	87.2% nylon 12.8% spandex (red)
			34	G2M17S1	90.2% nylon 9.8% spandex (sky blue)
			35	G2M18S1	84.4% nylon 15.6% spandex (black)
			36	G2M18S2	84.4% nylon 15.6% spandex (white)
			37	G2M19S1	65.8% nylon 17.7% spandex 16.5% polyester (beige)
			38	G2M20S1	89% nylon 11% spandex (blue)
			39	G2M21S1	86.1% nylon 13.9% spandex (black)
			40	G2M22S1	88.7% nylon 11.3% spandex (black)

gsm—grams per square meter.

**Table 3:** Results of thickness measurements of substrates in the preliminary experiment.

Type	Sample #1	Sample #2	Sample #3	Sample #4	Sample #5	Sample #6
No.	mm	mm	mm	mm	mm	mm
1	0.305	0.171	0.342	0.072	0.151	0.048
2	0.300	0.168	0.346	0.070	0.148	0.046
3	0.300	0.166	0.353	0.070	0.147	0.044
4	0.296	0.170	0.342	0.070	0.151	0.045
5	0.305	0.165	0.341	0.072	0.147	0.044
Average	0.301	0.168	0.345	0.071	0.149	0.045

**Table 4:** Results of thickness measurements of substrates in the main experiment.

No.	Index	Thickness	Average	No.	Index	Thickness	Average
–	–	mm	mm	–	–	mm	mm
1	G1M1S1	0.66	0.66	56	G1M12S1	3.32	3.25
2	G1M1S2	0.66		57	G1M12S2	3.26	
3	G1M1S3	0.66		58	G1M12S3	3.08	
4	G1M1S4	0.66		59	G1M12S4	3.28	
5	G1M1S5	0.65		60	G1M12S5	3.31	
6	G1M2S1	0.37	0.37	61	G1M13S1	3.38	3.42
7	G1M2S2	0.36		62	G1M13S2	3.32	
8	G1M2S3	0.37		63	G1M13S3	3.36	
9	G1M2S4	0.37		64	G1M13S4	3.44	
10	G1M2S5	0.37		65	G1M13S5	3.59	
11	G1M3S1	1.06	1.05	66	G1M14S1	4.76	4.66
12	G1M3S2	1.00		67	G1M14S2	4.81	
13	G1M3S3	1.04		68	G1M14S3	4.53	
14	G1M3S4	1.09		69	G1M14S4	4.60	
15	G1M3S5	1.06		70	G1M14S5	4.61	
16	G1M4S1	0.72	0.71	71	G1M15S1	4.13	4.15
17	G1M4S2	0.71		72	G1M15S2	4.08	
18	G1M4S3	0.70		73	G1M15S3	4.57	
19	G1M4S4	0.71		74	G1M15S4	4.02	
20	G1M4S5	0.71		75	G1M15S5	3.94	
21	G1M5S1	0.60	0.61	76	G1M16S1	4.90	4.79
22	G1M5S2	0.62		77	G1M16S2	4.81	
23	G1M5S3	0.61		78	G1M16S3	4.64	
24	G1M5S4	0.61		79	G1M16S4	4.76	
25	G1M5S5	0.62		80	G1M16S5	4.86	
26	G1M6S1	0.22	0.22	81	G2M1S1	0.83	
27	G1M6S2	0.22		82	G2M2S1	0.74	
28	G1M6S3	0.22		83	G2M3S1	0.82	
29	G1M6S4	0.21		84	G2M4S1	0.90	
30	G1M6S5	0.22		85	G2M5S1	1.06	
31	G1M7S1	0.72	0.72	86	G2M6S1	1.29	
32	G1M7S2	0.70		87	G2M7S1	0.59	
33	G1M7S3	0.71		88	G2M8S1	0.59	
34	G1M7S4	0.72		89	G2M9S1	0.66	
35	G1M7S5	0.73		90	G2M10S1	0.61	
36	G1M8S1	1.86	1.90	91	G2M11S1	0.75	
37	G1M8S2	1.94		92	G2M12S1	0.74	
38	G1M8S3	1.86		93	G2M13S1	0.56	
39	G1M8S4	1.96		94	G2M14S1	0.76	
40	G1M8S5	1.88		95	G2M14S2	0.77	
41	G1M9S1	2.03	1.96	96	G2M15S1	0.54	
42	G1M9S2	1.99		97	G2M16S1	0.66	
43	G1M9S3	1.85		98	G2M17S1	0.81	
44	G1M9S4	1.95		99	G2M18S1	0.63	
45	G1M9S5	1.99		100	G2M18S2	0.61	
46	G1M10S1	2.35	2.22	101	G2M19S1	0.85	
47	G1M10S2	2.29		102	G2M20S1	0.80	
48	G1M10S3	2.18		103	G2M21S1	0.55	
49	G1M10S4	2.14		104	G2M22S1	0.66	
50	G1M10S5	2.14					
51	G1M11S1	2.91	2.82				
52	G1M11S2	2.75					
53	G1M11S3	2.88					
54	G1M11S4	2.86					
55	G1M11S5	2.69					

**Table 5:** Pearson’s coefficient from measurements for light and strong pressure on substrate samples.

	Sample #1	Sample #2	Sample #3	Sample #4	Sample #5	Sample #6
***ε_r_***	0.815	0.964	0.939	0.998	0.992	0.997
**tg*δ***	0.881	0.957	0.933	0.986	0.940	0.959

**Table 6:** Average of value differences for light and strong pressure on substrate samples.

	Sample #1	Sample #2	Sample #3	Sample #4	Sample #5	Sample #6
***ε_r_***	0.08	0.12	0.088	0.09	0.18	0.06
**tg*δ***	0.0027	0.0073	0.0025	0.0067	0.0085	0.0078

**Table 7:** Average value of Pearson’s coefficient from measurements of *ε_r_* i tg*δ* in G1 and G2 groups of fabrics.

No.	Sample	*ε_r_*	tg*δ*
1	G1M1S*z*	0.988	0.981
2	G1M2S*z*	0.999	0.996
3	G1M3S*z*	0.998	0.942
4	G1M4S*z*	1.000	0.989
5	G1M5S*z*	0.994	0.996
6	G1M6S*z*	0.997	0.995
7	G1M7S*z*	1.000	0.988
8	G1M8S*z*	0.999	0.980
9	G1M9S*z*	0.999	0.981
10	G1M10S*z*	0.992	0.982
11	G1M11S*z*	1.000	0.968
12	G1M12S*z*	1.000	0.999
13	G1M13S*z*	1.000	0.980
14	G1M14S*z*	1.000	0.999
15	G1M15S*z*	1.000	0.996
16	G1M16S*z*	0.997	0.993
17	G2M1–22S*z*	0.763	0.460

**Table 8:** Selected parameters of RFID chip: NXP Ucode 7m (SL3S1214).

Parameter	Value
Sensitivity of reading	−16 dBm
Sensitivity of writing	−21 dBm
EPC memory	128 bit
User memory	32 bit
Input impedance @ 915 MHz	(12.8−j 248) Ω

**Table 9:** Measured dielectric parameters of DuPONT Pyralux LF9150R, at 866 MHz.

Substrate Thickness	*ε_r_*	tg*δ*
152 µm	2.855	0.110

**Table 10:** Numerical calculations of microelectronic module at 866 MHz.

Real Part of Impedance Re (*Z_TA_*)	Imaginary Part of Impedance Im (*Z_TA_*)	Power Transfer Coefficient *τ*
10.20 Ω	284.06 Ω	0.58

**Table 11:** Parameters of PACKLitzWire 10 × 0.04 (2 × 52) litz wire entered into the numerical model.

Number of Strands	Nominal Diameter	Total Diameter without Insulation	Total Diameter with Silk Insulation	Diameter of Double Silk Insulation
10	0.04 mm	0.186 mm	0.256 mm	0.070 mm

**Table 12:** Parameters of substrates considered in research.

No.	Sample	Average Value of *ε_r_*	Average Value of tg*δ*	Average Value of Thickness, mm
1	G1M1Sz	1.53	0.0051	0.66
2	G1M2Sz	1.78	0.0241	0.37
3	G1M3Sz	1.65	0.0001	1.05
4	G1M4Sz	1.75	0.0255	0.71
5	G1M5Sz	1.54	0.0123	0.61
6	G1M6Sz	1.62	0.0506	0.22
7	G1M7Sz	1.80	0.0333	0.72
8	G1M8Sz	1.84	0.0000	1.90
9	G1M9Sz	1.93	0.0000	1.96
10	G1M10Sz	1.51	0.0000	2.22
11	G1M11Sz	1.30	0.0000	2.82
12	G1M12Sz	1.35	0.0000	3.25
13	G1M13Sz	1.32	0.0000	3.42
14	G1M14Sz	1.42	0.0000	4.66
15	G1M15Sz	1.39	0.0000	4.15
16	G1M16Sz	1.55	0.0000	4.79
17	G2M1Sz	1.57	0.0015	0.83
18	G2M2Sz	1.60	0.0001	0.74
19	G2M3Sz	1.58	0.0001	0.82
20	G2M4Sz	1.72	0.0012	0.90
21	G2M5Sz	1.57	0.0000	1.06
22	G2M6Sz	1.75	0.0001	1.29
23	G2M7Sz	1.57	0.0049	0.59
24	G2M8Sz	1.62	0.0038	0.59
25	G2M9Sz	1.43	0.0004	0.66
26	G2M10Sz	1.43	0.0015	0.61
27	G2M11Sz	1.66	0.0043	0.75
28	G2M12Sz	1.24	0.0002	0.74
29	G2M13Sz	1.48	0.0002	0.56
30	G2M14Sz	1.37	0.0002	0.76
31	G2M15Sz	1.48	0.0001	0.54
32	G2M16Sz	1.53	0.0024	0.66
33	G2M17Sz	1.48	0.0026	0.81
34	G2M18Sz	1.66	0.0031	0.62
35	G2M19Sz	1.38	0.0001	0.85
36	G2M20Sz	1.36	0.0002	0.80
37	G2M21Sz	1.36	0.0002	0.55
38	G2M22Sz	1.80	0.0012	0.66

**Table 13:** Configuration of parameters of ISO/IEC 18000-63 protocol.

Parameter	Value
Forward link	DSB-ASK, Tari = 25 µs
Return link	FM0, 40 kHz
Command	Query

## Data Availability

All calculated and measured data will be provided upon request to the correspondent authors by email with appropriate justification.
